# Microglial Activation and Priming in Alzheimer’s Disease: State of the Art and Future Perspectives

**DOI:** 10.3390/ijms24010884

**Published:** 2023-01-03

**Authors:** Giulia Bivona, Matilda Iemmolo, Luisa Agnello, Bruna Lo Sasso, Caterina Maria Gambino, Rosaria Vincenza Giglio, Concetta Scazzone, Giulio Ghersi, Marcello Ciaccio

**Affiliations:** 1 Department of Biomedicine, Neurosciences and Advanced Diagnostics, Institute of Clinical Biochemistry, Clinical Molecular Medicine and Laboratory Medicine, University of Palermo, 90133 Palermo, Italy; 2 Department of Biological, Chemical and Pharmaceutical Sciences and Technologies (STEBICEF), University of Palermo, 90128 Palermo, Italy; 3 Department of Laboratory Medicine, University Hospital “P.Giaccone”, 90127 Palermo, Italy

**Keywords:** Alzheimer’s disease, microglia, phenotypes, activation, priming, β-Amyloid, central nervous system

## Abstract

Alzheimer’s Disease (AD) is the most common cause of dementia, having a remarkable social and healthcare burden worldwide. Amyloid β (Aβ) and protein Tau aggregates are disease hallmarks and key players in AD pathogenesis. However, it has been hypothesized that microglia can contribute to AD pathophysiology, as well. Microglia are CNS-resident immune cells belonging to the myeloid lineage of the innate arm of immunity. Under physiological conditions, microglia are in constant motion in order to carry on their housekeeping function, and they maintain an anti-inflammatory, quiescent state, with low expression of cytokines and no phagocytic activity. Upon various stimuli (debris, ATP, misfolded proteins, aggregates and pathogens), microglia acquire a phagocytic function and overexpress cytokine gene modules. This process is generally regarded as microglia activation and implies that the production of pro-inflammatory cytokines is counterbalanced by the synthesis and the release of anti-inflammatory molecules. This mechanism avoids excessive inflammatory response and inappropriate microglial activation, which causes tissue damage and brain homeostasis impairment. Once the pathogenic stimulus has been cleared, activated microglia return to the naïve, anti-inflammatory state. Upon repeated stimuli (as in the case of Aβ deposition in the early stage of AD), activated microglia shift toward a less protective, neurotoxic phenotype, known as “primed” microglia. The main characteristic of primed microglia is their lower capability to turn back toward the naïve, anti-inflammatory state, which makes these cells prone to chronic activation and favours chronic inflammation in the brain. Primed microglia have impaired defence capacity against injury and detrimental effects on the brain microenvironment. Additionally, priming has been associated with AD onset and progression and can represent a promising target for AD treatment strategies. Many factors (genetics, environmental factors, baseline inflammatory status of microglia, ageing) generate an aberrantly activated phenotype that undergoes priming easier and earlier than normally activated microglia do. Novel, promising targets for therapeutic strategies for AD have been sought in the field of microglia activation and, importantly, among those factors influencing the baseline status of these cells. The CX3CL1 pathway could be a valuable target treatment approach in AD, although preliminary findings from the studies in this field are controversial. The current review aims to summarize state of the art on the role of microglia dysfunction in AD pathogenesis and proposes biochemical pathways with possible targets for AD treatment.

## 1. Introduction

AD is one of the most common types of dementia worldwide, representing a major social burden with dramatic repercussions on patients, caregivers and the healthcare system. AD affects nearly 50 million people worldwide, and it has been estimated that it will double in European countries by 2050 [1]. Most AD cases are sporadic with the main risk factor being ageing. Disease hallmarks are Aβ extracellular deposits and intracellular hyperphosphorilated Tau aggregates, which drive neurodegeneration and atrophy within the cortex and hippocampus. Aβ derives from a precursor, Amyloid precursor protein (APP), which is cleaved by β and γ secretase enzyme in a multi step process. Broadly, it is accepted that Aβ deposition represents the *primum movens* in the pathogenesis of the disease because it triggers tau aggregation and inflammation in the brain.

Typical AD patients experience memory failure as the first manifestation of the disease, whereas atypical AD subjects present language problems. Either in typical or non-typical variants, AD patients progressively develop cognitive and neurobehavioral deficits and lose independence as regards daily activities, which distinguishes AD from mild cognitive impairment (MCI) [2]. AD symptoms derive from the loss of neurons throughout the brain cortex as a consequence of composite pathophysiological phenomena that had been first explained by the Amyloid cascade hypothesis. This assumed a direct, causative link between Aβ deposition and the pathophysiologic processes underlying AD, and originated from genetic evidence regarding the overexpression of the Aβ precursor protein (APP) gene in AD patients. Since other evidence supporting that hypothesis was obtained, Aβ has been supposed to be a good target therapeutic approach in AD treatment strategies. Monoclonal antibodies against various epitopes of Aβ have been developed and tested; however, no disease-modifying treatment has been reached, shedding light on the flaws of the Amyloid cascade hypothesis, from a pathogenic point of view. Currently, it is widely accepted that the classic AD hallmarks, Aβ aggregates and hyperphosphorilated Tau intracellular deposits, are insufficient to explain AD onset and progression, and the amyloid-inflammatory cascade has been recently formulated [3,4]. It posits that chronic microglia activation, following Aβ deposition, can initiate Tau protein alterations and accumulation in the brain [5,6,7]. Several observations have supported a role for microglial activation and priming in AD pathogenesis, including the correlation between plaque load and microglial activation in studies using animal models, the evidence that chronic activation of microglia during AD persists even with plaque burden reduction, the evidence that activated microglia surround plaques, the detection of elevated pro-inflammatory cytokine’s levels in AD patients, and, most importantly, the correlation of AD risk genes with native immunity [1]. In vivo and in vitro studies using different Aβ and Tau pathology models, along with two-photon spectroscopy studies, transcriptomic analyses and single cell mRNA sequencing studies have highlighted a possible role for microglia activation and priming in driving the progression of early to late AD.

According to the amyloid-inflammatory cascade, Aβ aggregate deposition first induces chronic microglia activation. Then, the latter leads to the “priming” of microglia. This promotes hyperphosphorylation of Tau protein and its intracellular deposition, with microscopic and clinical correlates [1,8].

Given this evidence, treatment strategies targeting microglial activation have been proposed, but disappointing results have been achieved. Recently, possible molecular targets for AD have been searched within those biochemical pathways that maintain microglia in a quiescent, neuroprotective state. A key player in this scenario is the neuronal-associated C-X-C motif chemokine ligand 1 (CX3CL1), which drives neuron-to-glia communication with the aim of keeping microglia in an anti-inflammatory, quiescent state (see “Microglia functions” paragraph-”Brain Homeostasis” subparagraph). CX3CL1 is a transmembrane protein normally expressed in neurons, presenting a complex molecular structure, with an N-terminal chemokine domain and a mucin-like stalk, that follows a short intra-cytoplasmatic domain. Throughout the central nervous system (CNS), CX3CL1 is mostly expressed in the hippocampus, where it interacts with its sole receptor CX3CR1, which is expressed by microglia and neurons. Once CX3CL1 binds its receptor, microglial activation is reduced through the inhibition of pro-inflammatory gene expression and cytokine (IL-1β, IL-6, and TNF-α) synthesis. This helps maintain the whole brain microenvironment in a quiescent/anti-inflammatory state, which is fundamental for neuronal progenitor cells (NPSs) of the neurogenic niche and for the brain homeostasis. It has been suggested that the disturbance of CX3CL1/CX3CR1 signalling could play a part in AD pathogenesis and progression [5]. Hence, the chemokine signalling has been included among those biochemical and inflammatory pathways and molecules that could represent a therapeutic target for AD treatment.

In this review, the role of microglia dysfunction and phenotypes in AD pathogenesis is presented, based on the recent literature, and the most attractive molecular targets for a novel AD therapy approach are reported.

## 2. Microglia in Health and Disease

Microglia are CNS-resident immune cells belonging to the myeloid lineage of the innate arm of immunity. They derive from yolk sac progenitors that migrate to the embryonic head and colonize the developing brain. Maintenance of the resident microglial population relies on a self-renewal mechanism and is independent of bone marrow haematopoiesis [9]. Until the early 2000s, microglia were known as a “static” cellular compartment, scaffolding the brain and displaying the capability of an immune response against pathogens, insults or cellular damage. Albeit true, this is profoundly incomplete. Microglia also exert “non-classical” activities as regards brain development and homeostasis, with substantial implications in neurodegenerative disease [10].

### 2.1. Microglia Functions

#### 2.1.1. Sensing

Microglia are in constant motion, extending and retracting their processes. This results in patrolling the CNS microenvironment to detect focal damage and provide for cellular homeostasis. Microglia scan the whole brain in a few hours, thanks to a transcriptomic apparatus called sensome, which allows the microglia sensing function [11,12,13]. Any function of microglia strictly depends on sensing, which allows the interaction between microglia processes and other brain cells. It has been substantiated that microglia continuously communicate with axons and dendritic spines of neurons and astrocytes [14]. This well-established phenomenon, called neuron-to-microglia communication, allows brain surveillance and drives homeostasis of CNS. Dysfunctional neuron-to-glia communication pathways are detrimental to cerebral health and cause disease [15]. Moreover, compromised sensing and surveillance have been associated with many diseases, including AD and Autism Spectrum Disorders (ASD) [5,16].

#### 2.1.2. Defence

Microglia sense pathogenic insult through their pattern recognition receptors (PRRs), detecting damage-associated molecular patterns and pathogen-associated molecular pattern (DAMPs and PAMPs, respectively) antigens [17]. Microglia activation through PRRs leads to face injury and repair damage. Notably, DAMPs include Aβ, meaning that microglia clear the oligomers of protein once they are produced and prevent its aggregation [18]. However, chronic activation resulting from repeated stimulation leads to detrimental effects. For instance, chronic Aβ deposition during AD leads to an aberrantly activated subset of these cells, which display a paradoxical neurotoxicity effect [5,18].

#### 2.1.3. Brain Homeostasis

Microglia guarantee brain homeostasis by ensuring effective synapse pruning and plasticity and maintaining myelin stability.

Microglia produce many cytokines and chemokines, including tumour necrosis factor (TNF), IL-1beta, IL-6, IL-8 and monocyte chemo-attractant protein 1 (MCP-1), in the absence of pathogenic stimulation. These cytokines normally influence the neural wiring of brain circuits during post-natal growth and adulthood [19]. TNF, one of the most studied, regulates the strength of connectivity in glutamate neural circuits by modulating the expression of AMPA receptors in the hippocampus. Beattie et al. reported that TNF-α increases the number of AMPA receptors and synapses, enhancing excitatory post-synaptic activity [20]. Moreover, TNF-α has been found to improve learning and memory in rats [21]. Although TNF-α is also produced by neurons, it has been proven that learning and memory formation strictly depend on microglia-derived TNF [22]. Finally, microglial cells have been shown to control brain plasticity processes, including nibbling and stripping of synaptic elements [23,24,25]. In a recent literature review, the effects of microglia on synaptic plasticity, learning and memory in healthy and pathologic conditions have been highlighted, concluding that dysfunctional microglia can contribute to impaired synaptic plasticity and cognitive functions during AD [26].

Microglia bind the chemokine CX3CL1, also called fractalkine (FKN) [21], through its CX3CR1 receptor. CX3CL1/CX3CR1 signalling allows continuous interplay between microglia and neurons, along with the CD200 signalling pathway [27,28]. Neuron-to-glia communication mediated by FKN pathways regulates learning and memory [29], as documented by Rogers et al., who reported impaired associative learning and hippocampal-dependent memory formation in CX3CR1−/− mice [30]. Furthermore, the expression of IL-1β was found to be increased in CX3CR1 knockouts, suggesting that CX3CL1 signalling lowers the production of IL-1β by microglia. An effect of CX3CL1 signalling on the neurogenesis of the adult has been demonstrated by in vivo studies. The CX3CL1/CX3CR1 pathway keeps the hippocampus microenvironment in a quiescent/anti-inflammatory state and protects neuronal progenitor cells (NPSs) of the neurogenic niche [30,31,32].

## 3. Microglia Phenotypes

Beyond the traditional and controversial description of microglia M1 and M2 subsets, currently many microglia phenotypes are known. All phenotypes present diverse morphology and molecular, metabolic and functional features, and it is widely accepted that different phenotypes correspond to diverse functions of microglia [33,34,35,36,37,38,39,40] (Table 1).

However, although the variety of microglia phenotypes is described, three main subsets of these cells can be defined to understand the role of microglia in the pathophysiology of AD. From a strictly functional perspective, microglia can act in the steady-state, the activated and the primed phenotype.

**Figure 1:**
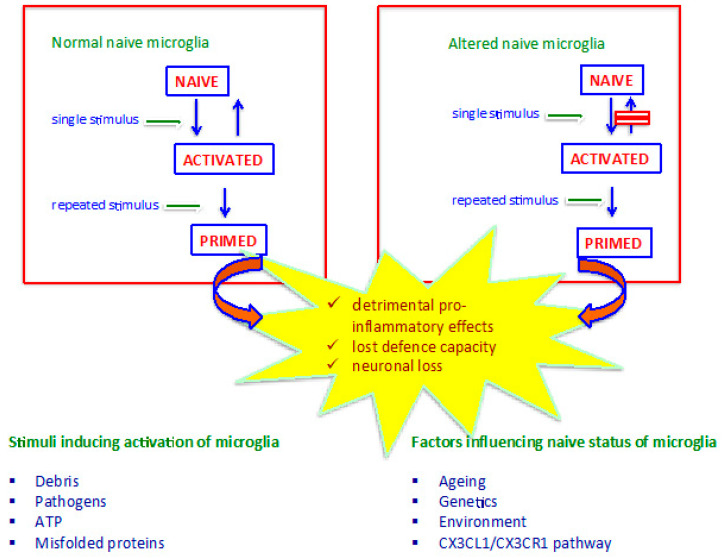
Microglia response upon stimuli should be effective and transient in order to face injury and avoid tissue damage deriving from excessive response. Once a stimulus activates microglia, they develop a protective, balanced response against injury, and then come back to the naïve state after the stimulus is eliminated. Upon repeated stimuli, microglia are primed via a toll-like receptor 4 (TLR-4)-mediated mechanism to enhance reactivity toward hit and pathogens. Both the efficacy and persistency of reactive microglia are influenced by the naïve, or baseline, steady-state status of these cells, which, in turn, is determined by a complexity of factors including genetics, ageing and neuron-to-glia communication. The latter maintains microglia in a quiescent, steady-state status, characterized by anti-inflammatory and neuroprotective behaviour. CX3CL1 is a critical modulator of neuron-to-glia interplay. Dysfunctional CX3CL1 signalling turns microglia toward a pro-inflammatory phenotype, resulting in an altered naive microglial status that tends to shift toward primed phenotypes earlier and easier than normal. Primed microglia emerge upon repeated stimuli and consist in microglia shifting towards less protective and more aggressive phenotypes, lacing the capacity to resolve inflammation and carrying out exclusively pro-inflammatory, detrimental effects. Primed microglia expose AD onset and progression and represent a promising target for a novel therapeutic approach in this disease.

Steady-state or naive microglia are always associated with neuroprotection, show a basic production of cytokines, and carry out housekeeping microglial activities [36,41]. Normally, naïve microglia are protective toward the brain microenvironment due to an anti-inflammatory asset. However, some conditions can lead steady-state microglia to display a less quiescent baseline phenotype, behaving more aggressively against injury and tending to prime easier than normal (see below). Activated microglia protect against pathogenic insult and injury, show enhanced production of cytokines, and are able to turn back to the naïve state once the stimulus has been removed. Activated microglia phenotypes overexpress pro-inflammatory IL-1β, IL-8, TNF and chemokines, which implies an aggressive and potentially harmful activity of these cells toward neurons. However, activated microglia also synthesize anti-inflammatory cytokines, which contribute to counterbalancing pro-inflammatory effects, thereby preventing sustained inflammation. Indeed, the key element of brain homeostasis is the balanced production of pro- and anti-inflammatory molecules by microglia, fine-tuning the response against stimuli [39,42]. When activated microglia are solicited by repeated stimulations, they orchestrate a heightened inflammatory response and display increased reactivity compared to the first stimulus [43]. Turning toward an aggressive, pro-inflammatory phenotype makes microglia “primed” to face challenges and fight injuries and infections adequately. Microglia priming is related to the ability of these cells to display memory against hit and pathogens and is a toll-like receptor 4 (TLR-4)-mediated mechanism [44]. Primed microglia never come back to the naïve state due to their resistance to regulatory feedback, and they have been associated with the pathogenesis of AD and other neurodegenerative diseases [45] (Figure 1) (Table 2).

The efforts to describe microglia phenotypes call attention to the core issues of this topic: which phenotype exposes to what disease, and how? Is it possible to prevent the onset or stop the progression from early to late AD by modulating microglia activation and priming?

It is apparent that aggressive, pro-inflammatory phenotypes make the brain prone to excessive inflammation, with a detrimental effect on neuron function and survival.

Many variables are known to interfere with the balance between protection and inflammation, including ageing, genetics, environment, and the baseline inflammatory state of the naïve phenotype (Figure 1). For instance, ageing-associated microglia perform ineffective immune surveillance due to fewer and shorter ramifications and display aggressive pro-inflammatory behaviour [46]. Primed microglia can be neurotoxic due to their pro-inflammatory behaviour generating a marked asymmetry between anti-inflammatory and pro-inflammatory balance in the brain microenvironment, which causes neuronal dysfunction and death.

Other factors affecting the balance between protection and inflammation include gender and neuro-protective molecules and the brain-derived neurotrophic factor (BDNF). Many studies have explored some sex-associated differences in microglia function, reporting a possible relation between these differences and several CNS diseases [47]. However, findings mostly regard CNS disorders other than AD, such as ischemia and traumatic injury, and they are still unclear.

BDNF sharply influences neurogenesis in the hippocampus, with evidence that decreased BDNF causes an imbalance of neurotrophic factors and subsequent alterations in the brain microenvironment in the early stages of the disease [48].

This article never proposes the word “neuroinflammation”, although the issue of inflammation in the brain is addressed. A controversy around the use of that term has been raised by some authors [14]. Actually, inflammation normally implies the recruitment and infiltration of immune cells at the site of injury, the expression of cytokines and other inflammatory products and their detectable blood levels. Oppositely, when activation of microglia occurs, inflammation in the brain is independent of recruited immune cells, no adaptive or innate peripheral immune cells infiltrate the brain, and no circulating cytokine levels can be detected during a microglial reaction. Post-mortem studies on AD human brains reported no perivascular mononuclear infiltrates or, generally, histopathologic features in AD brain tissue and in other neurodegenerative diseases, as well [49]. Importantly, cytokines are normally produced within the brain, as reported above, and their overexpression by microglia in response to injuries represents a normal event in the context of brain microenvironment homeostasis.

## 4. Microglial Role in AD

Evidence that microglia play a major role in determining AD comes from genome-wide association studies (GWAS), documenting that some genes strictly involved in microglia functions, including triggering receptors expressed in myeloid cells 2 (TREM2) and CD33, are associated with a fourfold higher risk for AD [50]. Different microglia-associated biochemical pathways have been studied in relation to AD development. Among all, TREM2, TYROBP, CD33, and C3 pathways have been deeply investigated, with a growing body of data supporting their potential contribution to AD pathogenesis [51,52,53]. TREM2 is a receptor-mediating inflammatory response in myeloid cells through the cooperation with the co-receptor TYROBP. TREM2 is known to regulate microglial activation, which is a two-step process that includes a TREM2-dependent step. Moreover, the impact of TREM2 on the Aβ response by microglia has been documented [51]. TYROBP is a transmembrane protein regulating the function of many microglia’s receptors, including CD33 and TREM2. TYROBP has been associated to protein aggregate deposition during AD and could play a part in the formation of DAM [52]. CD33 is an immunoglobulin-like lectin that is present on the surface of myeloid immune cells. It has been shown that microglia overexpress CD33 during AD, and inhibiting CD33 signal could have beneficial effects in AD brains [53]. Other studies have addressed the possible role of other microglia-basd mechanisms underlying AD development, including CR1, C1q, C3 and the complement cascade [11,54]. However, findings from the available studies are controversial or difficult to interpret, and, as such, should be taken with caution.

### 4.1. Microglia Activation and Aβ Pathology

Many mechanisms by which activated microglia are involved in AD pathogenesis are known, including failure in phagocytosis of Aβ, apoptosis-associated speck-like protein containing a C-terminal caspase recruitment domain (ASC) speck release, and the ageing-associated microglia features that make neurons prone to dysfunction and death. Additionally, a key player in the onset and progression of AD is priming, as noted above [5].

The phagocytic function is sustained by intracellular degradation through autophagy mechanisms. Modified proteins like Aβ and phosphorylated Tau aggregation compromise the functionality of the autophagocytic apparatus, leading to unfolded protein response in neurons [55]. Additionally, phagocytic function by microglia has been documented to be slow, thus making clearance of Aβ ineffective [56].

Further, microglia activation initiates a positive feedback loop, leading to Aβ species production enhancement. Activated microglia stimulate NF-kB, which induces the expression of NLRP3 inflammasome. This leads, in turn, to the release of ASC specks, which serve as core binding for Aβ aggregation [57,58]. Additionally, pro-inflammatory cytokines produced by activated microglia up-regulate the β-secretase (BACE) enzyme, which is the enzyme that cleaves the Aβ precursor APP. Activation of BACE increases Aβ species production [59].

Finally, aged microglia display unique characteristics that are associated with neurodegeneration and AD pathogenesis. First, they cover smaller brain areas with reduced immune-surveillance function due to fewer ramifications. This results in compromised sensing activity, which is associated with neurodegeneration. Further, aged microglia tend to maintain pro-inflammatory behaviour instead of balancing activation and resolution of inflammation phases [5].

### 4.2. Microglia Activation and Tau Pathology

As said before, the amyloid-inflammatory cascade hypothesis posits that microglia are the bridge linking Aβ deposition to hyperphosphorylated Tau aggregate formation. Aβ deposition represents a repeated stimulus that leads microglia to shift towards priming, losing their neuroprotective feature. Once microglia are primed, intracellular accumulation hyperphosphorylation of Tau takes place. An association between microglia activation and accumulation of Tau has been proven by many studies [45], and the interaction of Aβ, microglia and Tau has been substantiated by various pieces of evidence [5]. Tau has been documented to act as a stimulus for microglia activation and is able to induce overexpression of cytokines by microglia and changes in their phenotypes [60]. Further, Tau pathology-associated microglia show degeneration and impaired function [40]. Recently, Ising et al. suggested that the link between Aβ and Tau pathologies in AD is represented by NLRP3 inflammasome, and at least one signalling pathway interacting with both Aβ and Tau has been identified, which is the TREM2-TYROBP pathway [45,61]. In their analysis, Sekiya et al. demonstrate that the TREM2 pathway suppresses many signals induced by Aβ, showing a protective role of microglia, but enhances Tau pathology in a Tau TREM2-TYROBP model [61]. Similarly, the CX3CL1/CX3CR1 pathway interacts with both Aβ and Tau but elicits a ligand-dependent response, which is different upon FKN axis interaction with Aβ and Tau [62,63].

### 4.3. Microglia Activation and CX3CL1/CX3CR1 Signalling

Studies on animal models of Tau pathology and Aβ pathology have documented a role of CX3CL1/CX3CR1 signalling in the pathogenesis of AD, but controversial results have been achieved, with the FKN pathway being neurotoxic or neuroprotective depending on the mouse model used [64,65,66,67,68,69]. Hickman et al. analysed PS1-APP mice heterozygous for CX3CR1 (PS1-APP-CX3CR1+/−), which represent an AD-like Aβ pathology animal model. They reported an association of CX3CR1 deficiency with the reduction of Aβ levels, plaque burden and improved cognitive function [13]. Additionally, the authors documented an increase in PS1-APP-CX3CR1+/− mice of some molecules involved in Aβ degradation, including insulysin and matrix metalloproteinase 9, that are normally reduced in the brains of PS1-APP mice. The authors concluded that interfering with CX3CL1/CX3CR1 signalling could be an effective approach to increase neuronal Aβ clearance, reduce Aβ levels and delay progression of AD [13].

In their elegant experiment, Finneran et al. [63] isolated the chemokine domain and mucin-like stalk fragment of CX3CL1 and cloned into pTR2-MCS vector to produce mutant Tau Tg45 mice and littermate non-transgenic mice. The authors evaluated the impact of soluble CX3CL1 expression on cognition in tau rTg450 mice after the onset of cognitive deficits by intraparenchymal injections of FKN in the ventricular system through adeno-associated virus serotype 4. They documented that soluble FKN over-expression significantly improved cognitive performance, but did not reduce tau hyperphosphorylation.

Li et al. analysed an early stage disease APP/PS1 mice model using adenovirus-mediated gene transduction of bone marrow mesenchimal stem cells to deliver exogenous CX3CL1 and Wnt3a. They observed that exogenous CX3CL1 alone reduced the production of the inflammatory cytokines but did not improve cognitive function, while MSCs carrying CX3CL1 and Wnt3a ameliorated memory and learning [66]. Importantly, the authors demonstrated that the improvement of cognitive functions was related to the reduction of microglial neurotoxicity and increasing of hippocampal neurogenesis.

To summarize the findings from the studies on CX3CL1 axis in AD models, it can be stated that suppressing the CX3CL1 signal in hAPP mice and hTau mice causes increased tau phosphorylation and even exacerbates cognitive deficits, while in APP-PS1 and CRND8, it induces a reduction of Aβ deposition and microglial phagocytosis [15,70]. Experiments in 3xTg mice, displaying both Aβ and Tau pathology, reported that CX3CL1 deletion prevents neuronal loss [70]. These controversial results have been explained by the attitude of CX3CL1 signalling to keep microglia in an anti-inflammatory state, which reduces Aβ phagocytosis at the cost of increasing Tau phosphorylation. Many studies have been performed on the cerebrospinal fluid (CSF) of AD patients, reporting CX3CL1 levels to be elevated. However, contrarily, some authors have documented opposite findings [69,71].

From a molecular, biochemical and pathophysiologic perspective, it is reasonable to hypothesize that, in the very early stages of AD, dysfunctional (reduced) CX3CL1 signalling causes enhanced microglial phagocytosis and hyperphosphorylation of Tau protein [72]. These events sustain the unstoppable cascade of cellular and extracellular events and carry on the AD progression toward the late stages of the disease.

## 5. AD Treatment Strategies Associated with Inflammation and Microglia Activation

The efficacy of anti-inflammatory treatment strategies in AD has long been tested, but most of the attempts failed to prove a benefit, even slight, from NSAIDs, cytokines, and molecule-targeting transcription factors like NF-kB [5]. Results were disappointing with respect to certain concerns, including broad target (as is the case of NSAIDs) [73], difficulties in drug delivery (as regards anti-inflammatory cytokines) [74], and complications or hazards in the synthesis process of compounds (as per monoclonal antibodies against pro-inflammatory cytokines and molecule targeting transcription factors like NF-kB and p38MAPK) [75].

Recently, Lv et al. investigated Miltefosine, an anti-leishmanial drug activating the phosphatase magnesium-dependent 1A (PPM1A). In their study, the authors found that Miltefosine suppresses microglial NLRP3 inflammasome activation with beneficial effects in 3xTg mice [76]. Other authors achieved promising results by targeting NLRP3 inflammasome, modulating the release of IL-1β [77]. The advantage of these targets and molecules seems to be that they are well-tolerated, although further confirmation of their safety and efficacy is needed.

An allelic variant of the phospholipase C gamma 2 (PLCG2) gene, the P522R variant, has been recently investigated in AD, since PLCG2 is deemed to interfere with normal microglial function, contributing to the progression of the disease. Using CRISPR technology, Solomon et al. generated microglia derived from P522R heterozygous and homozygous cells. The authors found that heterozygous expression of the P522R variant resulted in increased microglial clearance of Aβ and preserved synapses. Moreover, upregulation of anti-inflammatory cytokine, including the Il-10 and CX3CR1, accompanied beneficial effects. However, the authors reported reduced synapse preservation when using cells that were homozygous for the variant, explaining this finding as a dose-dependent effect of P522RPLCG2 [78].

In a comprehensive review, Kann et al. suggest a pivotal role of IFN-γ in microglial priming, and propose targeting IFN-γ-activated microglia as a valuable target for AD treatment. Priming by IFN-γ results in proliferation, synapse elimination, and impaired synaptic transmission and cognitive functions. IFN-γ can shift microglia toward a neurotoxic phenotype once they are activated by a Toll-like receptor. Neurotoxic phenotypes of microglia, according to the authors, induce neuronal death through the release of oxidant compounds, and authors explained this event through a sort of control by adaptive immune cells over innate microglia [79].

Biochemical pathways influencing microglia through neuron-to-glia communication could be an optimal target, as suggested by previous authors [14,63,66]. Overall, the CX3CL1 axis could be an intriguing target, with some studies on in vivo and in vitro models achieving attractive, albeit preliminary findings [80,81,82].

## 6. Conclusions

Microglia housekeeping functions include sensing, immune surveillance and brain homeostasis maintenance, and depend on an anti-inflammatory, neuroprotective phenotype of these cells. At least one hundred genes for sensing are known, representing the so-called sensome. Neuron-to-glia communication maintains microglia in the neuroprotective, quiescent status, mainly thanks to the interaction between neuronal CX3CL1 and its microglial receptor CX3CR1. Given the importance of microglia functions and phenotypes in AD pathogenesis, it seems that novel targets for treatment strategies for AD could be sought among biochemical and molecular pathways regulating the housekeeping functions of these cells. It is hard to hypothesize an intervention on the sensome in this scenario, as considerable effort to identify target molecules among a multitude of genes is required. On the other hand, neuron-to-glia communication already represents an easy-to-explore biological context for these scopes. Particularly, preliminary results from studies on AD mouse models confirm the possible beneficial effect of CX3CL1 on ameliorating AD clinical features, although findings are preliminary and should be taken, as such, with a grain of salt. Moreover, investigations could address the issue of drug delivery, considering using technologies (nanotechnologies, for instance) to avoid a scenario in which a valuable target, but not the method for its administration in humans, is identified.

## Figures and Tables

**Table 1:** Main characteristics of different microglia phenotypes. Changes in microglia phenotypes occur depending on physiological and pathological conditions. CX3CR1: C-X-C motif chemokine ligand 1 receptor; Tmem119: transmembrane protein 119; P2ry12: P2Y receptors 12; P2ry13: P2Y receptors 13; DAM: Disease-associated Microglia; CNS: Central nervous System; TREM2: triggering receptor expressed in myeloid cells 2; TNFα: tumour necrosis factor α; iNOS: inducible nitric oxide synthase; CCL3: C-C motif ligand 3; CCL4: C-C motif ligand 4; CXCL16: C-X-C motif chemokine ligand.

Phenotype	Main Features
✓ Ramified microglia or steady-state, naïve phenotype	✓ homeostatic subset carrying out immune-surveillance and brain function regulation, expressing homeostatic genes (CX3CR1, Tmem119, P2ry12, P2ry13) ✓ neuroprotective
✓ Amoeboid-like microglia or proliferative region-associated phenotype	✓ first step of transition from naïve subtype to DAM; ✓ associated to CNS infections; ✓ shortened and thick ramifications, phagocytic activity; ✓ overexpress pro-inflammatory cytokines ✓ neuroprotective
✓ Rod-like microglia	✓ responsible for the detachement of pre-synaptic and post-synaptic membrane
✓ Dark Microglia (DA)	✓ associated to AD; preceeding Tau aggregation
✓ Disease-associated Microglia (DAM)	✓ Generated in two stages from homeostatic phenotype (step 2 involving TREM2); ✓ Expressing pro-inflammatory genes (TNFα, IL-1β, iNOS, IL-6, CCL3, CCL4, CXCL16) ✓ Increased cytokine production, phagocytosis and endocytosis; associated to AD, surrounding Aβ aggregates

**Table 2:** Differences between activation and priming give rise to microglia phenotypes that display profoundly diverse features, with consequences on brain microenvironment health and homeostasis. Due to their characteristics, primed microglia are associated with AD onset and progression. Altered baseline microglia, such as dystrophic microglia resulting from ageing or dysfunctional CX3CL1 signalling-associated microglia, increase the tendency of activated phenotype of these cells to shift toward the primed one.

Activation	Priming
✓ occurs upon a single stimulus ✓ not related to the immunological memory of innate cells	✓ after second or repeated stimuli ✓ related to the immunological memory of innate cells
✓ counterbalanced inflammatory response	✓ sustained inflammatory response
✓ comeback to naïve steady state	✓ no tendency to come back to the steady state
✓ maintaining defence capacity	✓ lost defence capacity
✓ causes neuroprotection	✓ causes neurotoxicity

## Data Availability

All data supporting opinions and conclusions reported in the current manuscript are available at pubmed.com; and scopus.com.
